# Working Memory Capacity as a Dynamic Process

**DOI:** 10.3389/fpsyg.2012.00567

**Published:** 2013-01-07

**Authors:** Vanessa R. Simmering, Sammy Perone

**Affiliations:** ^1^Department of Psychology, University of Wisconsin – MadisonMadison, WI, USA; ^2^Department of Psychology, University of Iowa and Delta CenterIowa City, IA, USA

**Keywords:** capacity, working memory, short-term memory, development, systems theory

## Abstract

A well-known characteristic of working memory (WM) is its limited capacity. The source of such limitations, however, is a continued point of debate. Developmental research is positioned to address this debate by jointly identifying the source(s) of limitations and the mechanism(s) underlying capacity increases. Here we provide a cross-domain survey of studies and theories of WM capacity development, which reveals a complex picture: dozens of studies from 50 papers show nearly universal increases in capacity estimates with age, but marked variation across studies, tasks, and domains. We argue that the full pattern of performance cannot be captured through traditional approaches emphasizing single causes, or even multiple separable causes, underlying capacity development. Rather, we consider WM capacity as a dynamic *process* that emerges from a unified cognitive system flexibly adapting to the context and demands of each task. We conclude by enumerating specific challenges for researchers and theorists that will need to be met in order to move our understanding forward.

## Introduction

Working memory (WM) has been dubbed the heart of intelligent behavior (Necka, 1992). One hallmark of the WM system is its highly limited capacity. WM capacity limitations are reliably associated with cognitive functions such as language comprehension, reasoning, planning, fluid intelligence, and scholastic achievement (e.g., Conway et al., 2003). Accordingly, capacity limitations are thought to influence cognitive development in a vast number of ways, from acquiring basic categories during infancy (Oakes et al., 2008), to higher-level skills such as following multi-step instructions (Gathercole et al., 2008). WM deficits have been observed in clinical populations, including children diagnosed with attention-deficit/hyperactivity disorder (ADHD; Willcutt et al., 2005), autism (Steele et al., 2007), developmental coordination disorder (Alloway, 2007), and schizophrenia (Cullen et al., 2010), as well as children born preterm (Vicari et al., 2004). Moreover, children with low WM scores (i.e., below the 10th percentile for age group on standardized assessment tasks) exhibit cognitive problems, symptoms of inattentiveness and distractibility, and difficulty with problem solving in academic settings (Alloway et al., 2009). WM capacity impacts cognitive functioning and development in multiple domains and populations. Understanding WM capacity development, therefore, has implications for our scientific understanding of human cognition and may enable researchers to foster positive changes in atypically developing populations.

How WM operates as part of a larger cognitive and behavioral system is highly complex. Facing such complexity, we must ask: What is our goal in studying WM capacity over development? One of the most obvious applications is to develop interventions that may alleviate the broad ranging negative consequences associated with poor WM (Alloway et al., 2009). Unfortunately, effective interventions have proven challenging to generate. For example, Shipstead et al. (2012) reviewed studies assessing whether training with the Cogmed program leads to improvements in WM and other symptoms of ADHD. Across studies, Shipstead et al. (2012) found mixed results, with some training effects failing to replicate, and overall more support for narrow versus broad transfer (an issue we discuss further below; for related discussion). We propose that a central limitation of previous training studies is that they are not motivated by theories of WM that specify how this system organizes itself across contexts.

To design effective interventions, we must understand the cognitive processes and behaviors we study. In this way, we argue, our theories are failing us: through decades of research we have not yet developed a robust approach to improving WM capacity, much less reliably influencing the related behavioral consequences. As an attempt to help our science move beyond its current limitations, we review data and theory on WM capacity development, evaluate limitations of existing theoretical approaches, and propose that understanding the cognitive and behavioral processes we measure in the laboratory requires a deeper appreciation for the complexity of the system we study. The empirical database on WM capacity development has grown immensely during the past half century. Despite this growth, much remains to be understood about the mechanisms that underlie developmental changes in WM capacity (Cowan, 2010). In fact, one could argue that such this growth has painted an unclear picture of children’s performance in WM tasks. We propose that the time has come to rethink existing approaches to WM capacity and its development.

The impetus to reconsider our approaches to studying WM capacity is motivated by our survey of developmental changes in WM capacity. This survey highlighted key challenges to our understanding of WM capacity development, which we discuss in further detail below. Briefly, we found that capacity estimates^1^ increase reliably over development in nearly every study, but vary substantially across tasks and domains when compared within age groups. Furthermore, tasks designed to tax WM more (by adding manipulation or processing demands) or less (by requiring only storage) do not result in straight-forward modulations of capacity. These facts raised five key questions that we will address in this paper, which we summarize briefly here.

### Do different tasks simply “tap” WM capacity differently?

We will show that WM capacity for children of the same age varies substantially across task contexts. One view of such cross-task variation in performance is that different tasks tap WM capacity differently. We disagree: in our view, capacity does not exist as a separable property of the cognitive system and therefore cannot be “tapped.” Instead, we view cross-task variation in performance as a signature of a dynamically self-organizing cognitive and behavioral system.

### Might task differences mask a child’s underlying WM capacity?

Historically, WM capacity has been viewed as a competency that an individual possesses with improvement over development. From this view, task differences can mask children’s underlying competency. In our view, task and stimulus differences are external influences on the dynamic organization of the cognitive and behavioral systems involved in performing the task at hand. Indeed, we will review our own work showing that the number of items maintained in memory actually changes across contexts. We reject the competence-performance distinction because, in our view, competence cannot be measured and is therefore an unfalsifiable construct.

### Can we only understand WM capacity development by using the same task across age groups?

The immense growth of the literature on WM capacity development has led to the use of numerous tasks and variations of the same task across research groups. This, in turn, has created the substantial variation in children’s capacity estimates reported and an unclear picture of the trajectory along which capacity develops, revealing how complex the task of understanding capacity development is. At first glimpse, these facts might suggest that understanding children’s behavior over development would be simplified by merely using the same task with multiple age groups. We will show, by contrast, that use of multiple tasks within an age group illuminates the dynamics of the cognitive and behavioral systems we study. Thus, we suggest that measuring the WM system in multiple contexts is more informative than assessing the same context across multiple time points in development.

### Can theories incorporating multiple, simultaneously developing abilities account for WM capacity development?

We will describe several theories of WM capacity development that attribute change to a single mechanistic source. We suggest that such views are unable to simultaneously account for the cross-task variation in children’s performance and universal increase in capacity estimates. If various single mechanisms are unable to provide an account of children’s performance in WM tasks and development, might the addition of several, simultaneously developing abilities do the job? For example, might improvements over development in both rehearsal and resistance to interference provide an adequate account of increases in capacity estimates? In our view, the answer is no. Too often, such theories still treat these cognitive abilities as separable and independent. We view performance in WM tasks as determined by multiple, reciprocally coupled cognitive and behavioral processes that are not linearly separable.

### Are existing theories simply underspecified?

We will argue that existing theories cannot simultaneously account for the cross-task variation in children’s performance and the universal increase in capacity estimates. Might increased specificity enable existing theories to overcome their limitations? In our view, existing theories of WM capacity are not simply underspecified. Moreover, we view existing theories as qualitatively different from the types of theories needed to understand WM capacity and its development. We advocate a new way of thinking about what capacity is, how capacity develops, and our global approach to studying capacity.

### Overview

This paper is divided into three main sections. In the first section, we present a survey of developmental changes in WM capacity in visual and verbal domains (for more in-depth reviews, see Dempster, 1981; Jenkins et al., 1999; Cowan et al., 2007). We then review theoretical explanations of WM capacity development, which have historically attributed changes in capacity to single, or separable, mechanistic source(s). Such explanations have limited applicability across tasks and domains. Our second section outlines our proposal that focusing on single mechanistic sources – or even multiple, separable sources – of WM capacity limits and its developmental change cannot explain cross-context variation. Alternatively, we propose that a systems approach is best suited for understanding both general improvements in WM capacity over development and specific variation across contexts at a given time point. In this section, we present two case studies illustrating one systems approach to WM capacity development. In our third section, we discuss the challenges we believe theories and empirical inquires of WM capacity must meet in order to acquire a deeper scientific understanding of WM processes and ultimately develop interventions that foster positive cognitive outcomes for individuals suffering from WM deficits.

## Working Memory Capacity Development

### Survey of empirical results

Working memory is typically divided into separate verbal and visuo-spatial subsystems, each with a limited capacity (see Cowan, 2001, and commentaries for in-depth discussion). Within each domain, some researchers draw a distinction between tasks measuring short-term versus WM, with the former requiring only storage and the latter requiring additional manipulation of information (for discussion, see Cowan, 2008). However, this boundary is not uniform, with some theorists drawing no distinction between the two (e.g., Heyes et al., 2012). The application of the terms “short-term” and “working” memory have been inconsistent within the WM literature and, indeed, may not be separable processes. Here, we focus on comparing tasks according to similar methodology, but consider all tasks to fall under the umbrella term “WM” (see Appendix for methodological details and exclusions). More specifically, Table 1 shows a broad sample of studies using “simple span” tasks (i.e., requiring only storage) to assess verbal WM capacity. Table 2 shows the same for visuo-spatial WM capacity. Table 3 shows results from one common type of “complex span” task, specifically backward span in which participants are required to repeat stimuli in the opposite order from encoding. Within Table 3 we separate results by verbal versus visuo-spatial stimuli for ease of comparison with Tables 1 and 2. Lastly, Table 4 includes results from studies using other types of complex span tasks, such as those requiring further manipulation of information (e.g., counting objects to arrive at the digits to be remembered) or dual-tasks (e.g., serial recall of auditory digits while ignoring visually presented letters). Included in these tables is a survey of nearly 200 tasks and conditions from multiple studies across 50 papers that stimulated our rethinking of WM capacity and its development.

**Table 1:** **Estimates of verbal working memory capacity using simple span tasks**.

	Age (years)													
	2	3	4	5	6	7	8	9	10	11	12	13	14	15
Bayliss et al. (2005a)
Digit recall						[	4.3	]					
Bayliss et al. (2005b)
Digit recall				[		4.0			]			
Case et al. (1982)														
Word recall		3.0	3.7	4.5	4.5									
Cowan et al. (2005)														
Auditory span recognition							2.8		3.3		4.0			
Digit recall^a^							4.7		4.9		5.4			
Cowan et al. (2006a)														
Digit recall^a^							5.7							
Cowan et al. (2006b)														
Digit recall (auditory)									4.0					
Letter recall (visual)									3.2					
Cowan et al. (1999)														
Digit pretest					[	5.4	]	[	6.4	]			
Attended digits^b^					[	4.0	]	[	4.8	]			
Cowan et al. (2006c)														
Name span (visually presented)							4.8			5.6				
DeMarie and Ferron (2003)														
Word span				3.2	3.8		3.8	3.7	4.1					
Digit span				4.2	4.4		4.8	5.0	5.1					
Dempster (1981)^c^														
Digit recall	2.3			4.4		5.0		6			6.5			
Letter recall				3.5		4.5		4.9			5.3			
Word recall	3.0			4		4.2		4.3			4.5			
Engle and Marshall (1983)														
Digit recall, slow presentation					3.5				4.7					
Digit recall, fast presentation					4.3				6.1					
Gilchrist et al. (2009)														
Word recall, order irrelevant						4.0		5.2						
Halford et al. (1994)														
Digit recall^b^				4.2			[ 5.1 ]			6.1			
Hulme et al. (1984)														
Short word recall^b^			2.0			3.0	3.0		3.5	4.0				
Medium word recall^b^			1.5			2.1	2.2		3.0	3.1				
Long word recall^b^			0.7			1.2	1.3		1.9	1.9				
Huttenlocher and Burke (1976)														
Digit recall, grouped			3.1			4.3		5.4		5.7				
Digit recall, ungrouped			2.3			3.0		3.9		4.6				
Hutton and Towse (2001)														
Digit recall							4.8			5.4				
Imbo and Vandierendonck (2007)														
Digit recall									5.5	5.8	5.7			
Isaacs and Vargha-Khadem (1989)														
Digit recall						5.2	5.7	5.8	6.1	6.1	5.8	6.2	6.3	6.7
Miller and Vernon (1996)														
Tone sequence recall			[	2.9	]								
Morra and Camba (2009)														
Digit recall							[	5.3		]	
Nichelli et al. (2001)														
Word span^a^				[ 4.3 ]		4.2	5.1	5.1	5.8	6.1	6.5			
Noël (2009)														
Word recall			3.5	3.8										
Orsini et al. (1981)														
Digits^a^				3.5	3.7	4.2	4.5	4.9	5.0	5.1				
Ottem et al. (2007)														
Similar word recall		2.8	3.0	3.0	3.4	3.4	3.4^a^	3.6	3.8	3.8	3.8^a^	3.9	3.9	3.8
Distinct word recall		3.0	3.4	3.6	3.5	3.6	3.6^a^	3.8	3.8	4.0	4.2^a^	4.1	4.3	4.0
Pickering et al. (1998)														
Letter recall					3.2			4.0						
Russell et al. (1996)														
Short word recall, verbal report					4.0									
Short word recall, pointing					3.8									
Long word recall, verbal report					3.4									
Long word recall, pointing					3.4									
Wynn and Coolidge (2009)														
Digit recall														6.1
Zuber et al. (2009)														
Letter recall					[	4.3	]						

*Brackets indicate data derived from age groups spanning more than 1 year. For complete details of which experiments and conditions were included from each paper, as well as specifics of mean ages and ranges and full citations, see Appendix*.

*^a^These results are averaged across experiments, conditions, or groups presented within the same paper, as comparable methods were used*.

*^b^Precise values were not presented in text; therefore, estimates are derived from the data figure(s) presented*.

*^c^These values are estimated from reviews of multiple other studies; see reference for details*.

**Table 2:** **Estimates of visuo-spatial working memory capacity using simple span tasks**.

	Age (years)														
	3	4	5	6	7	8	9	10	11	12	13	14	15	16	17
Bayliss et al. (2005a)															
Corsi block recall					[	4.5	]							
Bayliss et al. (2005b)															
Corsi block recall			[		4.2			]					
Chi (1977)															
Familiar faces, layout preserved^a^			1.8												
Familiar faces, layout irrelevant^a^			3.9												
Unfamiliar faces, layout preserved^a^			1.1												
Unfamiliar faces, layout irrelevant^a^			3.5												
Cowan et al. (2005)															
Color array recognition^b^						3.6		4.4		4.8					
Cowan et al. (2006b)															
Letter recall (visual)								3.2							
Color array recognition (one item cued)								2.4							
Cowan et al. (2006c)															
Spatial span recall (no repetitions)						5.6			6.7						
Spatial span recall (with repetitions)						4.8			5.7						
de Ribaupierre and Bailleux (1994)															
Pattern recall			1.4	1.8	2.2	2.5	2.9	3.4	3.8	4.2	4.2	4.4			
de Ribaupierre et al. (1989)															
Pattern recall			1.4	1.6	2.1	2.6	3.1	3.3							
Hamilton et al. (2003)															
Visual span recall, study 1			[	4.5	]	[	6.8	]	[	9	]				
Visual span recall, study 2 control				[	3.5		]								
Spatial span recall, study 1			[	3.5	]	[	4.5	]	[	5	]				
Spatial span recall, study 2 control				[	2.8		]								
Hitch et al. (1988)															
*Experiment 1*															
Line drawings, similar			1.4					3.6							
(Named)			2.5												
Line drawings, long names			1.8					2.8							
(Named)			2.6												
Line drawings, control			2.1					3.7							
(Named)			2.7												
*Experiment 2*															
Line drawings, control (named)^a^			1.3					3.5							
Hitch et al. (1989)															
Visually similar drawings			1.4						3.4						
Phonemically similar drawings			2.0						2.7						
Dissimilar drawings			1.9						3.6						
Isaacs and Vargha-Khadem (1989)															
Corsi block recall					4.1	4.7	4.7	5.3	5.3	5.4	5.4	5.4	5.6		
Kyttälä (2008)															
Pattern recall, students with math problems													[ 7.2]		
Pattern recall, students with math and reading problems													[ 6.5]		
Pattern recall, control students													[ 8.3]		
Corsi block recall, students with math problems													[ 5.3]		
Corsi block recall, students with math and reading problems													[ 5.3]		
Corsi block recall, control students													[ 6.3]		
Picture recognition, students with math problems													[ 5.1]	
Picture recognition, students with math and reading problems													[ 4.9]	
Picture recognition, control students													[ 6.2]	
Logie and Pearson (1997)															
Corsi block recall^a^			2.8			4.5			5.2						
Corsi block recognition^a^			3.7			7.1			8.0						
Pattern recall^a^			3.0			6.2			8.0						
Pattern recognition^a^			4.0			10.9			14.8						
Luciana et al. (2005)															
Spatial span							[ 5.1]		[ 5.8]		[ 6.6]			[ 6.5]	
Miller and Vernon (1996)															
Color sequence recall		[	3.6	]											
Color array recall		[	4.7	]											
Shape sequence recall		[	3.3	]											
Shape array recall		[	4.6	]											
Morra and Camba (2009)															
Pattern recall							[	3.7		]					
Mutter et al. (2006)															
Pattern recall	0.8	1.3	1.9												
Nichelli et al. (2001)															
Spatial span (Corsi)			[ 3.4]		3.8	4.2	4.4	4.7	4.6	5.1					
Noël (2009)															
Corsi block recall		2.7	3.5												
Orsini et al. (1981)															
Corsi block^b^			2.7	3.1	3.9	3.9	4.2	4.3	4.5						
Palmer (2000)															
*Verbal recoding condition (Experiment 1)*															
Picture series recall	1.8			2.4	2.8										
Phonologically similar	1.7			2.3	2.1										
Visually similar	1.6			2.0	2.4										
*Silent/control condition (Experiment 1)*															
Picture series recall	1.8			2.4	2.7										
Phonologically similar	1.7			2.2	2.3										
Visually similar	1.7			2.1	2.4										
*Longitudinal, labeling not controlled (Experiment 2)^c^*															
Picture series recall			2.3	2.7	3.4	3.7									
Phonologically similar			2.3	2.4	2.5	2.5									
Visually similar			2.0	2.3	2.8	3.2									
Pickering et al. (1998)															
Block recall			2.8			3.2									
Riggs et al. (2006)															
Color array recognition			1.5		2.9		3.8								
Simmering (2012)															
Color array recognition	1.9	2.2	2.8		3.8										
Vicari et al. (2003)															
Complex figure spana			2.4	2.7	2.8	3.0	3.3	3.4							
Corsi block recall^a^			3.1	3.8	4.3	4.6	4.7	4.8							
Wagner and Jackson (2006)															
Picture recognition^a^				3.0	3.0		5.4								
Wilson et al. (1987)															
Pattern recognition, 2s delay^a^			3.7		8.1				14.1						
Pattern recognition, 10 s delay^a^			2.8		6.9				10.9						
Zuber et al. (2009)															
Corsi block recall				[	4.1	]									

*Brackets indicate data derived from age groups spanning more than 1 year. For complete details of which experiments and conditions were included from each paper, as well as specifics of mean ages and ranges and full citations, see Appendix*.

*^a^Precise values were not presented in text; therefore, estimates are derived from the data figure presented*.

*^b^These results are averaged across two experiments presented in the same paper*.

*^c^Two cohorts of children completed the task at three time points in this experiment, beginning at ages 5 and 6 years; the groups did not differ when compared at the same age, so results for 6 and 7 years are averaged across these groups*.

**Table 3:** **Estimates of working memory capacity using backward span tasks**.

	Age (years)														
	3	4	5	6	7	8	9	10	11	12	13	14	15	16	17
**VERBAL**															
Hutton and Towse (2001)															
Digit recall						3.8			4.6						
Isaacs and Vargha-Khadem (1989)															
Digit recall					3.2	3.6	3.8	3.8	4.5	4.3	4.4	4.0	4.8		
Morra (1994)															
Digit recall^a^				2.6	3.1	3.3	3.7	4.1							
Word recall				2.5	3.1	3.3	3.5	3.9							
Morra and Camba (2009)															
Digit recall						[	4.5		]						
Wynn and Coolidge (2009)															
Digit recall													[	4.3	]
Zuber et al. (2009)															
Letter recall				[	2.5	]									
**VISUO-SPATIAL**															
Hitch et al. (1988)															
Line drawings, control (named)^b^			1.7					3.0							
Isaacs and Vargha-Khadem (1989)															
Corsi block recall					3.9	4.5	4.7	4.6	5.1	5.0	5.4	5.2	5.5		
Luciana et al. (2005)															
Spatial span							[ 5.0]		[ 5.5]		[	5.9	]	[6.4 ]	
Zuber et al. (2009)															
Corsi block recall				[	2.9	]									

*Brackets indicate data derived from age groups spanning more than 1 year. Results reported here were from backward versions of tasks reported in Table 1 (listed under Verbal) and Table 2 (listed under Visuo-Spatial). For complete details of which experiments and conditions were included from each paper, as well as specifics of mean ages and ranges and full citations, see Appendix*.

*^a^These results are averaged across two experiments presented in the same paper*.

*^b^Precise values were not presented in text; therefore, estimates are derived from the data figure presented*.

**Table 4:** **Estimates of working memory capacity using complex and/or dual-tasks**.

	Age (years)														
	3	4	5	6	7	8	9	10	11	12	13	14	15	16	17
Bayliss et al. (2005a)															
Verbal-verbal					[	3.7	]								
Verbal-visuo-spatial					[	3.1	]								
Visuo-spatial-verbal					[	4.4	]								
Visuo-spatial-visuo-spatial					[	3.1	]								
Bayliss et al. (2005b)															
Verbal-verbal				[		3.1			]						
Visuo-spatial-visuo-spatial				[		3.8			]						
Case et al. (1982)															
Counting span recall				1.7	2.3	2.5	3.0	2.8	3.2	3.5					
Cowan et al. (2011)															
Shape-color-location recognition															
Silent trials^a^				[	1.9		]		[	2.5	]				
Name color trials^a^				[	2.4		]		[	3.0	]				
Say “wait” trials^a^				[	1.5		]		[	2.1	]				
Cowan et al. (2005)															
Counting span recall^b^						2.8		3.6		4.0					
Listening (sentence) span recall^b^						1.8		2.5		2.7					
Running (digit) span recall^b^						2.1		2.6		2.7					
Ignored speech (digits) recall						2.0		2.0							
Cowan et al. (2006b)															
Digit (auditory) and letter (visual) recall															
Attend auditory								3.6							
Attend visual								1.7							
Ignore auditory								3.4							
Ignore visual								1.7							
Cowan et al. (2010:1]															
Shape-color-location recognition; two shapes															
1-Shape^a^					[1.5 ]				[1.9 ]						
100% Trials^a^					[1.3 ]				[1.8 ]						
80% Trials^a^					[0.8 ]				[1.5 ]						
50% Trials^a^					[0.5 ]				[1.3 ]						
20% Trials^a^					[0.4 ]				[1.0 ]						
Shape-color-location recognition; three shapes															
1-Shape^a^					[1.5 ]				[2.5 ]						
100% Trials^a^					[1.3 ]				[2.2 ]						
80% Trials^a^					[0.5 ]				[1.5 ]						
50% Trials^a^					[0.5 ]				[1.0 ]						
20% Trials^a^					[0.4 ]				[0.8 ]						
Cowan et al. (1999)															
Unattended digits (visual dual-task)^a^				[	2.4	]	[	3.1	]						
Cowan et al. (2006c)															
Counting span recall						3.6			4.1						
Running span recall						2.1			2.8						
de Ribaupierre and Bailleux (1994)															
Color/location recall			1.1	1.4	1.7	1.8	2.0	2.5	2.8	3.1	2.9	3.0			
de Ribaupierre et al. (1989)															
Color/location recall		0.6	1.1	1.3	1.6	1.9	2.4	2.6							
Halford et al. (1994)															
*Experiment 1*															
Word order, subtraction dual-task							3.3								
Word order, reading dual-task							3.6								
*Experiment 2*															
Digit recall, integrated condition						[	3.1		]						
Digit recall, non-integrated condition						[	3.2		]						
*Experiment 3*															
Digit recall, counting dual-task^a^			1.8				2.7			3.8					
*Experiment 4*															
Digit recall, forward counting dual-task						2.2				2.8					
Digit recall, backward counting dual-task						1.9				2.7					
Digit recall, story listening dual-task						3.4				2.6					
Hutton and Towse (2001)															
Digit recall, forward with suppression						3.7			4.5						
Digit recall, backward with suppression						3.5			4.2						
Counting recall, forward						3.3			4.6						
Counting recall, backward						2.4			3.6						
Morra (1994)															
Counting span recall^b^				2.1	2.4	2.7	3.1	3.8							
Color/location recall^b^				2.0	2.5	2.7	3.2	3.9							
Morra and Camba (2009)															
Counting recall						[	3.4		]						
Russell et al. (1996)															
Counting span recall (simple)				4.4											
Counting span recall (complex)				3.6											
Odd-man-out position recall (simple)				4.2											
Odd-man-out position recall (complex)				3.7											
Sum span recall (simple)				4.2											
Sum span recall (complex)				3.2											
Towse and Hitch (1995)															
Counting span recall (features)^a^				2.8	3.5	4.7		4.2							
Counting span recall (conjunction)^a^				2.6	2.7	3.3		3.6							
Counting span recall (feature-slow)^a^				2.6	2.8	3.3		3.8							
Towse et al. (1998)															
Counting array recall (small first)^a^				2.6	2.7		4.1	4.1							
Counting array recall (large first)^a^				2.3	2.5		3.7	3.9							
Operation span recall (short)^a^^,^^b^							3.3	3.9	3.8	4.4					
Operation span recall (long)^a^^,^^b^						2.5	3.4	2.9	3.5						
Reading span recall (short)^a^						2.9	2.9	3.2							
Reading span recall (long)^a^						2.2	2.7	2.9							
Towse et al. (2002)															
*Short interpolation task (6 s)*															
Operation span recall, parity dual-task^a^									2.0						
Operation span recall, accuracy dual-task^a^									2.2						
Operation span recall, parity, and accuracy^a^									1.9						
*Long interpolation task (15 s)*															
Operation span recall, parity dual-task^a^									1.7						
Operation span recall, accuracy dual-task^a^									1.8						
Operation span recall, parity, and accuracy^a^									1.2						

*Brackets indicate data derived from age groups spanning more than 1 year. Tasks are not divided between verbal and visuo-spatial domains because many included components from each system. For complete details of which experiments and conditions were included from each paper, as well as specifics of mean ages and ranges and full citations, see Appendix*.

*^a^Precise values were not presented in text; therefore, estimates are derived from the data figure presented*.

*^b^These results are averaged across two experiments presented in the same paper*.

One striking similarity across studies and domains is the almost universal increase in capacity estimates over development. Within nearly every study, capacity estimates are higher for older children than younger children. This strongly suggests a pervasive developmental change that manifests in a variety of tasks that estimate capacity in different ways. In contrast to the reliable increases in capacity estimates described above, however, these studies also illustrate that capacity estimates vary substantially across tasks and domains *within a single age group*. For example, consider 7-year-olds performing simple verbal tasks (Table 1): capacity estimates across 18 studies range from as low as 1.2 (Hulme et al., 1984) to as high as 5.4 items (Cowan et al., 1999). Even when comparing memory for only one type of stimuli – digits – the range still spans from 3.0 (Huttenlocher and Burke, 1976) to 5.4 (Cowan et al., 1999). Comparing 7-year-olds’ performance in simple visuo-spatial tasks (Table 2) shows another 27 studies or conditions with a range of 2.1 (Palmer, 2000) to 8.1 items (Wilson et al., 1987). This variability indicates substantial influence of task details on capacity estimates even for paradigms designed to assess storage alone.

When considering tasks designed to assess manipulation of information, results from 7-year-olds’ performance in backward span tasks (Table 3) are entirely within the range from forward tasks (albeit at the low end): for letters, 2.5 (Zuber et al., 2009) to 3.1 (Morra, 1994); for digits, 3.1 (Morra, 1994) to 3.2 (Isaacs and Vargha-Khadem, 1989); and for blocks in the Corsi task, 2.9 (Zuber et al., 2009) to 3.9 (Isaacs and Vargha-Khadem, 1989). One might argue that backward spans are more informative when compared to forward span within a single group of participants. Isaacs and Vargha-Khadem did such a comparison, testing the same children in both forward and backward versions of both digit and Corsi block tasks (see Tables 1– 3). They found that performance differed significantly based on order for digits only, not for blocks in the Corsi task. This suggests that the influence of manipulating order in memory also depends on the details of the task.

Potential sources of developmental change in WM capacity are no less puzzling when examining performance in complex tasks. Consider children’s performance in counting span tasks, the most common complex task variant in Table 4. For 6- and 7-year-olds, capacity estimates range from 1.7 (Case et al., 1982) to 4.4 (Russell et al., 1996) digits in this task, which is slightly lower than the range in Table 1. Interestingly, multiple studies showed that small differences in how stimuli were presented (e.g., in a canonical versus random spatial organization; Russell et al., 1996) influenced capacity estimates in this paradigm. Looking later in development, capacity estimates for 11- to 12-year-olds (still considering digits in the counting span task) range from 3.2 (Case et al., 1982) to 4.6 (Hutton and Towse, 2001), again showing variation based on stimulus presentation. What can we conclude from this range of findings? If we assume that complex tasks require processing or manipulation above and beyond the storage required in simple tasks, then we would (presumably) expect lower estimates from complex tasks than simple tasks. This is the case if we consider means across studies, but not necessarily the case if we consider the full range of estimates.

The results summarized in Tables 1– 4 raise a critical question: what do we strive to explain with our theories? Do we want to explain differences in means between age groups or differences across tasks within age groups? Is it more important to understand why some simple tasks yield lower estimates than some complex tasks? As we will discuss below, one limitation of current theories is they do not yet address such cross-task and -age variation. This is a critical limitation because complex tasks such as these have long been held as measuring individual differences in adults’ cognitive ability and as robust predictors of reading comprehension (Daneman and Carpenter, 1980), performance on standardized tests (Turner and Engle, 1989), and problem solving (Engle et al., 1999). Furthermore, Cowan et al. (2005) showed that both simple and complex task measures correlate with scholastic performance during middle childhood.

In summary, the goal of the research presented in these tables has been to map the developmental trajectory of WM capacity. However, achieving this goal has proven a daunting task. In fact, the vast literature on WM capacity development has created an unprecedented theoretical challenge: to explain the WM processes that underlie the universal increase in capacity estimates *and* how the very same WM processes produce variation in capacity estimates across tasks and domains. In the next section, we describe current and historical theoretical perspectives on WM capacity development.

### Theories of WM capacity development

Providing a concise synthesis of theories of WM capacity development is not easy. One key obstacle is that theories in this domain have been closely tied to specific periods of development, specific domains, and/or the specific tasks they address. For example, there is currently a heated debate between “slots” and “resources” theories in research on visual WM capacity (e.g., Alvarez and Cavanagh, 2004; Bays and Husain, 2008; Zhang and Luck, 2008). However, these theories have thus far only been applied to memory for continuously varying visual stimuli (e.g., color, orientation) in simple tasks (recall or recognition of small arrays) with adults (but, see Heyes et al., 2012, for one developmental study in this debate), making it unclear how such theories would address the range of tasks and age groups shown in Table 2. Theories rarely, if ever, make contact with a broad range of tasks, domains, or developmental periods. In our synthesis, we discuss a few select theories based, in part, on their prevalence, historical significance, and their scope. Our aim was to present a review of the most comprehensive and compelling theories of development that have been put forth, rather than an extensive review of many competing theories.

The majority of theories of capacity development have focused on the universal increase in capacity estimates, rather than the variation in performance across tasks and domains. Many theorists have proposed that developmental increases in WM capacity arise from improvements in cognitive processes other than WM capacity such as processing speed, attention, encoding, response selection and generation, and/or retrieval (see, e.g., Towse and Hitch, 2007, for further discussion). For example, Case (1995) proposed that developmental improvements in performance reflected changes in processing speed and efficiency, which eventually lead to more information being stored with the same capacity/mental resources. He considered children’s experience to be a driving force behind improvements in processing. This feature of Case’s perspective could potentially account for differences across tasks, stimuli, and individuals. For example, as a child gains experience with counting, she/he may become more efficient at processing digits and therefore have an increased digit span, but not improve in spans of non-digit words. Indeed, Fry and Hale (1996) showed that measures of processing speed in non-span tasks account for much of the variance in capacity estimates (derived from storage plus processing tasks) both within and between age groups. The developmental hypothesis from this perspective, then, is that improvements in other cognitive processes enable the WM system to exhibit an increased capacity in laboratory tasks without any change in mental resources.

Dempster (1981) also proposed that increases in WM capacity estimates arise from sources other than capacity. Dempster classified 10 factors that lead to individual and developmental differences in capacity into two categories: four were strategic variables, and six were non-strategic variables. Although many researchers propose that strategic variables (e.g., rehearsal, chunking) are a primary source of developmental improvement in WM tasks, there is little direct evidence regarding how or why strategies might change over development, and Dempster concluded that the evidence at that time was not strong enough to support these types of explanations. For non-strategic variables (e.g., speed of processing, resistance to interference), on the other hand, theorists have put forth some mechanisms that may produce developmental change. In particular, Pascual-Leone (1970) and Case (1995) propose that mental resources, referred to as M-space or M-power, are changing over development. Some researchers suggest that M-space is increasing over development, whereas others believe that the efficiency with which M-space is used is the source of developmental improvement. In either case, the primary mechanism proposed to cause this developmental change is an increase in myelination. According to Case, this is a likely source because myelination not only increases the speed of neural conductivity, but also reduces interference through insulation. Although it seems intuitive that these changes would produce either an increase in resources available or improve the efficiency of the use of these resources, no specific process has been proposed by which increased neural conductivity would improve performance in span tasks.

Most early theoretical explanations of WM capacity development attribute increases in capacity estimates to a single cause (e.g., those reviewed by Dempster, 1981). One more recent example of this type of approach was illustrated by Oberauer and Kliegl (2001). These authors were interested in potential sources of aging-related declines in WM capacity. They discussed five potential sources of WM capacity limitations (limited resources, fixed slot-like capacity, speed of decay versus rehearsal, similarity-based interference, and inter-item confusion) and developed computational models to test whether these sources of WM capacity limitations could account for data comparing young and old adults’ performance on a complex WM task. They tested seven formal models that implemented these sources (three types of resource models and one model for each additional proposed source) and found that only two models – one with capacity limits arising through decay, the other arising through interference – provided a good fit to their data. This approach assumes that there is only one source of capacity limits. Can we assume that WM capacity would be limited by *either* decay or interference, but not both? Similarly, should we infer that only one of these would change over development?

As the aforementioned examples illustrate, single-cause explanations have fallen short in accounting for the type of variation across tasks and domains shown in Tables 1– 4. For example, although Fry and Hale (1996) showed that processing speed accounts for much of the variance they found in WM tasks, there was still a significant proportion of variance from age that was *not* accounted for by speed. Thus, WM performance increased over development due to some process(es) *in addition* to processing speed. The limitations of such single-cause explanations has led theorists to argue for explanations that incorporate multiple causes (e.g., Towse and Hitch, 2007; Cowan, 2010), suggesting the need for studies that control and compare multiple processes as well as storage constraints. Cowan et al. (2010), for example, highlights the distinction between processing-related and storage-specific limitations addressed through previous research. He argues that processing strategies (such as rehearsal, chunking, or imagery) are likely to vary across task contexts whereas storage limits stay constant across a wider variety of circumstances. He proposes that future research should aim to control or prevent processing strategies to gain a more precise measure of storage.

Similarly, Towse and Hitch (2007) outlined evidence for four sources of variation in WM performance: increases in processing speed, improved ability to maintain information while engaged in a separate processing task, increased storage capacity, and improved response-timing processes (e.g., latency to respond, pauses between items). They further proposed that developmental changes in performance arise from the combination of which processes are accessed and how well they function. We agree that theories must move beyond single-source models of WM capacity limits and development. However, our perspective makes the further claim that multiple sources of developmental differences cannot be considered in isolation. Below, we propose a new view of WM capacity that embraces the complex manner in which neural, cognitive, and behavioral systems interact over real and developmental time scales. We believe this view can provide new insights into the sources of both variation and stability across development. We provide evidence from two case studies that have tested novel predictions derived from this perspective.

## A New View on Working Memory Capacity Development

Our proposal is that WM capacity and its development can best be understood as a dynamical system, from the perspective of a systems theory. A key feature of systems theories is their emphasis on process over representation (Thelen and Smith, 1994). Capacity has long been studied as a concrete property of the WM system: a given individual is considered to “have” a capacity in each domain, which is then brought to bear on laboratory tasks. Indeed, the fact that Tables 1– 4 present numerical estimates of how many items are held in memory points to this emphasis on representation. From a systems perspective, by contrast, the processes of remembering cannot be separated from what is being remembered (i.e., representation; Spencer and Schöner, 2003). We contend that WM capacity does not exist in the way it has historically been discussed: from our perspective, there is no such thing as capacity “in the head.” Rather, capacity estimates are *emergent* products of cognitive and behavioral systems interacting in real-time in a task context. Emergence – a central concept in systems theory – is the notion that a given property of a system exists only through the meeting of components of that system. To draw on an analogy from physics, consider the concept of friction, which exists solely as the product of the meeting of two surfaces: a ramp alone has no friction, as a ball alone has no friction, but rolling a ball down a ramp produces friction. Extending this to WM, capacity does not exist alone, but rather capacity estimates are an emergent product of various components of cognitive and behavioral systems organizing themselves over time within a specific stimulus and task context.

Additionally, capacity estimates arise from the nature of the coupling between cognitive and behavioral systems. Coupling – another central concept in systems theory – is the notion that the dynamics of one system depend on and are inseparable from the dynamics of another system. Consider the classic example of two pendulum clocks (Rosenblum and Pikovsky, 2003). In the seventeenth century, Huygens (1986) observed the spontaneous synchronization of the motion of two clocks. How did this happen? Both clocks were connected to the same wooden beam. The motion of the clocks generated vibration that was shared across the two clocks. The common source of energy – coupling – led the behavior of the two clocks to become perfectly synchronized without the need for any outside source of intervention. That is, the behavior of the two clocks was the product of a self-organizing dynamical system that emerged from the coupling between the two systems.

In our view, capacity in the classic sense (e.g., the “slots” metaphor) does not work. In the laboratory, we derive capacity estimates that are the emergent product of multiple, highly complex, coupled cognitive and behavioral systems operating within the task context. If we want to understand why capacity estimates appear limited and why they differ across individuals, development, and task contexts we must understand the *dynamics* of these systems (i.e., how the components of a system interact through time). We illustrate this claim below by reviewing two case studies from our own work. Our proposal stands in contrast to the historical approach to understanding capacity and its development. For instance, Cowan (2010) emphasized the role of processing (e.g., strategy) in explaining cross-task performance differences, while contending that storage remains relatively constant across tasks. Though we agree that both processing and storage must be considered to understand performance across tasks, we disagree with both the characterization of storage as a separable component of the system as well as the notion that storage is constant across tasks. *In our view, storage capacity cannot be “tapped.”* Storage is a process in and of itself that cannot be considered in isolation from the processes that contribute to (e.g., encoding, chunking) and operate upon (e.g., rehearsal, retrieval) stored information.

Below, we present two case studies illustrating how a systems approach can be applied to WM capacity development. These studies have tested specific predictions derived from the implementation of visual WM into a computational model, which allows for direct testing of how changes in a given set of processes may simulate developmental improvements in performance. These examples demonstrate how the specific details of the behavioral tasks designed to measure WM capacity influence the processes by which WM representations are formed and used in service of the tasks, and reveal that capacity may vary *within the same participants* depending on the manner in which information is presented and capacity is measured. Importantly, we do not consider these differences across tasks to be “noise” in our estimates, but rather believe this cross-task variation informs our understanding of how this dynamic cognitive and behavioral system operates and develops.

### Case study 1: Infant visual working memory

**Figure 1:**
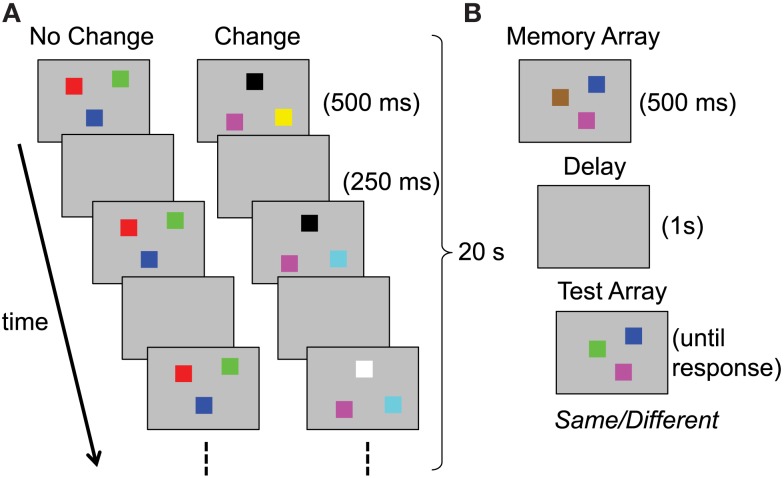
**Schematic illustrations of tasks used to assess visual working memory in (A) infants versus (B) children and adults; both present examples of set size three**.

Our first case study centers on a series of neural network simulations reported by Perone et al. (2011). Perone et al. showed that a single, complex system can produce remarkable variation in performance across contexts. More specifically, they tested the prediction that a single neuro-dynamical systems model of infant looking and memory could produce variation in infants’ capacity estimates across task conditions. They simulated infants’ performance in a change preference task designed by Ross-Sheehy et al. (2003) to estimate visual WM capacity. Figure 1A shows this task, in which infants viewed two displays of colored squares blinking on and off in synchrony. On a “no-change” display, all of the colors remained the same with each blink/delay. On a “change” display, one randomly selected color changed to a new color. Infants’ looking time to the two displays was compared, and a robust preference for the change display was interpreted as memory for the number of items per display (i.e., set size). Across set sizes, Ross-Sheehy et al. found that 6-month-olds showed a robust change preference only at set size one, whereas 10-month-olds showed change preferences up to set size four. They concluded that infants’ visual WM capacity increases from one to four items between 6 and 10 months.

Perone et al. (2011) simulated infants’ performance in this task using a model of infant looking and memory. The model consists of a neurocognitive system that encodes object details (e.g., color) and a fixation system that is biased to sustain looking during encoding. Encoding leads to WM formation of the colors in the displays; once a robust WM is formed, inhibition biases the system to look away from remembered items and explore items that may be novel. The model exhibited a change preference through recognition of the items on the no-change display *and* detection of novelty on the change display. This preference emerged through real-time interactions between looking, encoding, and WM formation. Critically, Perone et al. found that a preference for the change display *did not require memory for all items in the display*, that is, the model exhibited a higher capacity estimate (measured through looking time) than the number of items maintained in WM.

This example highlights how multiple processes working together give rise to behavioral estimates of capacity. Critically, the challenge remains to understand how such processes give rise to variation in performance like that shown in Tables 1– 4. Within systems approaches, such variation is viewed as a signature of a system that organizes in real-time in response to the current task context. Perone et al. (2011) illustrated this concept by simulating a second experiment by Ross-Sheehy et al. (2003) in which they removed the delay to insure that young infants’ performance reflected a limitation in memory, not perception or attention. Indeed, young infants exhibited change preferences for set sizes up to three in this condition. This manipulation changed the task in two important ways. First, “blinks” on the change and no-change displays were no longer present, that is, there were no transient onsets within each presentation of the items. Second, it introduced a “flicker” associated only with the changing item on the change display. Perone et al. showed that these minor manipulations dramatically influenced looking behavior. In the DNF model, looking and memory are reciprocally coupled components of a larger cognitive and behavioral system. Manipulations of looking influenced memory formation. In particular, the “flicker” associated with the change display biased the model to preferentially look to the display. This had two consequences. First, it led the model to encode the changing item on the display, which, in turn, biased the model to look at the change display more. Second, it enabled the model to encode more items into WM on the change display, boosting the number of items that the model maintained. Performance in memory tasks reflects the real-time organization of a system in context rather than a property of the WM system.

Critically, this no-delay version of the change preference task does not depend on memory in the classic sense (i.e., maintaining information across a delay). Nevertheless, Perone et al. (2011) simulations showed that the same cognitive and behavioral system that captured infants’ performance in task contexts that involved a delay could also capture infants’ performance in a task context with no-delay. This illustrates the emergence of capacity in our perspective: in the no-delay condition, support from the task input allowed for more items to be encoded and maintained in memory. This, in turn, facilitated recognition of sameness on the no-change display and detection of novelty on the change display. The result was a robust preference for the change display. In contrast, the standard condition is less supportive and places a different burden on the memory system. The repeated delay slows memory formation for non-changing items and promotes the decay of items maintained in memory. This, in turn, led to a different pattern of looking by the model. This example highlights how a task context impacts the process of remembering, the number of items remembered, and capacity estimates.

In light of the data in Tables 1– 4, a critical question is whether such a system is able to produce both behavioral variability across contexts as well as stable developmental increases in capacity estimates. As illustrated by our simulations of the no-delay condition, in a dynamical system real-time behavior emerges via the interactions of its components in context. The tendency of a dynamical system to produce similar behaviors in the same context (i.e., stability) depends on the nature of interactions among its components (for an illustration and discussion, see Perone and Spencer, 2012). Thus, developmental change in stability requires modification in the interactions among components. Perone et al. (2011) proposed that older infants’ change preferences at higher set sizes emerged from changes in the strength of interaction among the excitatory and inhibitory neural components governing encoding and memory formation in their model. Stronger interactions have a variety of emergent consequences, including faster and more robust WM formation, stronger suppression of encoding via WM, and increased responsiveness to novelty. This impacts how cognitive and behavioral processes interact in real-time. Perone et al. showed that the same model with stronger interactions performed like older infants (i.e., exhibited higher capacity estimates), maintaining multiple items on the no-change display while encoding novelty on, and looking longer to, the change display.

### Case study 2: Visual working memory through childhood into adulthood

Simmering (2008), under review) used a similar model architecture to address inconsistent estimates of visual WM capacity over development. As described above, Ross-Sheehy et al. (2003) estimated capacity at one item for 6-month-olds, increasing rapidly to an adult-like three to four items by 10 months of age. By contrast, studies with older children using the change detection task revealed estimates of only one to two items during the preschool years, not reaching adult levels of performance until adolescence (see Table 2; Cowan et al., 2005; Riggs et al., 2006; Simmering, 2012). In this task, a small number of colored squares are presented briefly (e.g., 500 ms), followed by a short delay (e.g., 1 s) and presentation of a test array that includes either all of the same colors, or one new color (see Figure 1B). Participants are instructed to respond *different* if any items changed, or *same* if all items were unchanged. One obvious source of the inconsistent estimates between infancy and later childhood is the different tasks used to assess capacity. Without a full understanding of the processes involved in these tasks, however, it was not possible to relate performance across the two tasks and development (see Riggs et al., 2006, for discussion).

Simmering (2008) addressed this inconsistency by implementing both tasks within the same neural network model, and then testing one group of participants (3-, 4-, 5-year-olds, and adults) in both tasks to compare performance directly. Model simulations showed how the same memory processes could operate in both tasks but yield strikingly different capacity estimates. In particular, the repeated presentation of items on both displays in the infant task allowed WM representations to build incrementally, which was particularly important for younger children, whose memory builds more slowly and is less stable. By contrast, successful performance in the change detection task requires robust WM representations to form quickly and be maintained stably enough to support generation of a *same/different* decision.

These simulations led Simmering (2008) to predict that capacity estimates should be higher in the change preference versus change detection task when measured in the same participants, but, critically, that performance should still be correlated across tasks due to the shared processes of WM formation and maintenance. Behavioral results confirmed these predictions, showing capacity estimates from the change preference task of at least six items for all age groups, but change detection capacity estimates of only two to three items for children and four to five items for adults. Moreover, change detection capacity was predicted by a behavioral signature of robust memory formation in looking tasks: the number of times participants looked back and forth (Kovack-Lesh et al., 2008; Perone and Spencer, 2012). In particular, the number of times participants looked back and forth between displays on set size four trials was positively correlated with capacity, even after controlling for age-related improvements (see Simmering, 2008, for details).

Following these behavioral results, Simmering (2008) quantitatively fit the model’s performance to children’s and adults’ results from both tasks, in order to understand how the same memory system could produce such different estimates across tasks. The model was indeed able to maintain more items in WM during the change preference versus change detection task. As such, Simmering concluded that the infant task did not over-estimate capacity, but actually *produced higher capacity* than the change detection task. The same model showing different capacity – not just estimates, but the actual number of items held in WM – across tasks illustrates the benefit of conceptualizing capacity *as a* *process*. Returning to the issues we raised at the beginning of this paper, alternative interpretations of these results could be that the looking task reveals a competence that is masked by the change detection task, or that the tasks tap different underlying memory systems. Combining the empirical and computational results, however, support our position that the system organizes in the task context: storage cannot be separated from the processes that form and act upon representations, and storage limits are not static across task contexts.

## Challenges for Theories of Working Memory Capacity Development

The empirical results in Tables 1– 4 illustrate the complex pattern of WM capacity development: although capacity increases universally, different stimuli, paradigms, and domains have revealed high variability in estimates that remains to be explained. Until recently, the dominant theories of WM capacity development focused on single-cause explanations for capacity limits and their developmental increase, which we contend are unable to capture the full range of behavior shown in Tables 1– 4. There has already been a move to theories that consider multiple causal factors (e.g., Towse and Hitch, 2007; Cowan et al., 2010), but such theories characterize processes and storage as separable cognitive components. As an alternative, we believe that successful theories of WM capacity and development will re-conceptualize capacity as part of the *process* involved in performing various tasks rather than a separable, static storage component. Our case studies illustrate how this new approach can shed light on previously unclear behavioral results. We are not arguing that existing theories are simply underspecified. We are arguing for a re-conceptualization of what capacity estimates truly measure.

We have identified three challenges that we believe theories must confront in order to provide a comprehensive account of WM capacity and its development. Our position is that meeting these challenges will allow our theories to overcome previous limitations and extend our theories beyond the tasks we use in the lab to realize positive impacts on real world behaviors that relate to WM capacity.

### Challenge 1: System components must be specified

A central challenge for any theory of WM capacity is to postulate the components of the cognitive and behavioral systems under study, and to specify the coupling among those components. This is necessary to understand the real-time organization of the system in the behavioral tasks we use in the laboratory. Tables 1– 4 give *estimates* of capacity, which researchers derived through some calculation based on *behavior*. Across different tasks, the specific behaviors we measure can differ substantially (e.g., serial reproduction of verbal lists, judging each item in a list as old/new, yes/no comparisons of multi-item visual arrays, pointing to remembered locations), but we expect these diverse measures to reveal some common underlying cognitive mechanisms. Consider, for example, our second case study in which looking behavior in the change preference task was compared to *same/different* judgments in the change detection task. Without a formal proposal regarding the contribution of the items being held in memory versus the response processes, there would be no foundation for the predicted relationship between participants’ looking behavior and their capacity estimates.

By incorporating the specific details of the task designs and behaviors we measure in the laboratory into our theories, we can begin to understand how common cognitive and behavioral processes could produce the wide variety of estimates shown in Tables 1– 4. For example, it is generally accepted that capacity estimates are influenced by attention, processing speed, and inhibition (e.g., Johnson et al., 2003). How multiple, interacting processes organize themselves in service of behavior, however, is strongly influenced by the context. High attentional demands may lead to lower capacity estimates in one study while simpler stimuli lead to higher capacity estimates in another. To understand WM capacity and its development, we must simultaneously consider multiple contributions to behavior and the capacity estimated from it.

Our case studies above illustrate how such specification may be achieved through the use of computational models. Implementing multiple behavioral tasks within a single model architecture requires specifying what the components are, how they are coupled, and how they interact. This can lead to novel behavioral predictions. For example, in addition to the case studies we described above, Simmering and Patterson (2012) showed how the developmental mechanism proposed by Simmering (2008) to capture visual WM capacity development also predicts improvements in color discrimination in a single item memory task. Note that we are not proposing that computational models are necessary for a rigorous approach to understanding the link between cognition and behavior; rather, we emphasize keeping a tight link between theories and experiments with particular attention on real-time behaviors.

### Challenge 2: Theories must be linked closely to real-time behavior

Theories of WM capacity and its development cannot evolve without a tight connection between theory and experiment. The work of Karmiloff-Smith and colleagues provides several elegant examples (from other domains of cognitive development) of how this may be accomplished without the use of computational models. For example, Paterson et al. (2006) assessed numerical skills across populations by testing multiple age groups with multiple behavioral tasks. Their results showed that assumptions about infant processing based on adult dissociations were not supported. Rather, understanding the cognitive and behavioral processes involved in each task at each point in development revealed how processing styles diverged between groups over development. This example illustrates how non-computational approaches can still achieve the necessary level of specificity to explain behavior across multiple tasks and populations, and that new insights can be gained by considering cognitive and behavioral processes *in context*.

Although there will always be a place in our science for the types of measures reported in Tables 1– 4, we believe new methods of analysis hold great promise in providing new insights into developmental changes into cognitive processes. One example of this type of approach across domains is to consider “micro-behaviors” – that is, behavioral indices of underlying cognitive processes that can be found in the fine details of real-time behavior – rather than macro-level measures like percent correct. In the context of our own case studies, we have demonstrated how looking *dynamics* (i.e., switching between displays) may be a more sensitive measure of capacity than looking *preference* alone. But even this measure is relatively coarse, and does not tell us about the temporal unfolding of switches and duration of each fixation across the course of a trial. These measures might be informative about the link between online cognitive processing and behavior.

Taking advantage of the richness of behavioral data promises to drive the study of WM capacity forward. This approach has led to new insights into infant habituation. For example, Perone and Spencer (2012) showed that the duration of looks at a stimulus early in learning structured the time course of memory formation. This, in turn, influenced the duration of looks later in learning. The details of fixation, then, provide meaningful information about the underlying process of forming a memory and how memory formation impacts fixation dynamics, above and beyond classic measures associated with habituation. In work with adults, Spivey (2007) has demonstrated how the real-time dynamics of decision making (measured through movements of the eyes and/or computer mouse in lab tasks) reveal probabilistic representations when overt behavioral measures (e.g., which item on the screen the participant clicked) could be interpreted as deterministic. This emphasis on the fine details of behavior presents a challenge to researchers because it will require new ways of collecting and analyzing data.

### Challenge 3: Integration across types of stimuli – verbal and visuo-spatial – and tasks – simple and complex

Historically, the separation of verbal and visuo-spatial memory systems has been widely accepted. At the very least, visual WM tasks typically require looking at images or events (e.g., colored squares, hand movements to blocks) and verbal WM tasks typically require reading or hearing stimuli (e.g., words, letters, digits). Critically, however, Tables 1– 3 show that visual and verbal WM can both be characterized by a universal increase in capacity estimates and cross-task variation in capacity estimates. Should we interpret this as parallel changes in unrelated systems, or evidence for shared processes across different types of stimuli? Can we reunite verbal and visual subsystems?

From a systems view, much of this work will be done by specifying the relevant components of the system (as described in Challenge 1). For example, many theories posit separate storage for verbal versus visuo-spatial information, but shared attentional mechanisms that influence encoding into these stores (e.g., Cowan et al., 2005). However, as illustrated by our example above of Isaacs and Vargha-Khadem’s (1989) study of backward digit and Corsi spans, it seems that this relationship will not be straight-forward: their results suggested that the process of manipulating item order was not comparable for verbal versus visuo-spatial stimuli. As we argue above, the processes that act upon stored information cannot be considered separately from the information being stored.

A related concern is where (and whether) to draw the line separating simple and complex tasks. The typical argument for this distinction emphasizes the need for processes other than storage in complex tasks. However, across the range of studies in Tables 1 and 2, we found that participants were required to encode and retrieve information in a variety of ways across tasks. As such, even “simple” tasks depend on more than just storage. Although these differences across tasks are relatively small compared to the processes involved in, for example, a counting span task, we argue that it is still informative to consider these tasks as arising from the same unified system: demands on some processes may be minimized in simple tasks, but these processes still exist and contribute to performance.

One compelling argument to consider simple and complex tasks separately is raised by Engle and colleagues (e.g., Shipstead et al., 2010). They emphasize that simple tasks do not correlate consistently with performance outside the lab, and suggests that simple tasks are therefore less informative for understanding individual differences and interventions. If we consider simple and complex tasks as part of the same self-organizing system, however, then we could use the differences between the task types as an indication of the processes that are most critical to measures of general intelligence. For example, Unsworth and Engle (2007) showed that performance on supraspan trials of simple tasks is maximally predictive of general fluid intelligence, whereas performance on complex tasks is maximally predictive at small spans. To us, this suggests that both types of tasks are dependent upon the same underlying system which organizes differently according to context. How this organization occurs in response to the task context may be just as important to understanding individual differences and/or interventions as understanding how the system operates in each context separately.

### Rising to the challenge: What is the goal of our theories?

What do we want, or *need*, to achieve with theories of WM capacity? To date, our theories have often been limited to specific tasks, domains, and developmental periods. Might there be an advantage to develop theories with a broader goal in mind? We think the answer is yes. We contend that developing theories with broader goals will ultimately provide us with a deeper scientific understanding of the systems we study. Moreover, we contend that developing theories with broader goals will have practical implications in the real world. Consider WM training programs as one example. These involve intensive exercises designed to improve WM capacity. The target outcome of such programs is to improve cognitive functioning in the real world by reducing memory lapses, improve scholastic achievement, limit symptoms for those suffering from ADHD, and even increase intelligence (for review of the claimed benefits of one particular training program, see Shipstead et al., 2012).

Within the past few years, numerous reviews of WM training programs have emerged, highlighting this as a central goal of research on WM capacity (e.g., Shipstead et al., 2010; Melby-Lervåg and Hulme, 2012; Wass et al., 2012). Many of these reviews have reached the pessimistic conclusion that the evidence for WM training that achieves the intended broad, positive impact on cognitive function is mixed at best. What is the source of this pessimism? Our survey of Shipstead et al. (2012) review of the Cogmed WM training program and the related commentaries points to the central assumption underlying the development of such programs: capacity is a *property* of the WM system (for discussion, see Logie, 2012; see also Klingberg, 2012). If WM capacity can be enhanced, then individuals should exhibit benefits across the wide array of contexts that this property operates within. However, the evidence indicates that WM training has limited transfer, with the primary benefit on tasks that closely resemble the training tasks. Indeed, some have argued that improved performance on such tasks may arise from nothing more than familiarity with the task or even performing tasks on computers, and is not an effect of WM capacity at all (Hulme and Melby-Lervåg, 2012).

Even more worrisome is the fact that our current theories do not reliably predict which tasks will show transfer of WM training. Our view echoes that of Gibson et al. (2012), that our theories and WM training studies must inform each other. Moreover, we must have theoretically motivated research that specifies how WM training impacts the WM system and how the WM system is integrated with other cognitive and behavioral systems across contexts (Hulme and Melby-Lervåg, 2012; Shipstead et al., 2012). Finally, we must move our theories beyond a single task, domain, and developmental period in order to explain – and predict – how WM changes within an individual over time (Gibson et al., 2012). If we do not understand how the tasks we use in the lab relate to one another, we will not be able to predict when training in lab tasks will produce lasting improvements in behaviors outside of the lab.

## Conclusion

Working memory capacity plays an important role in numerous cognitive functions and continuously serves behavior in the real world. Limitations in WM capacity impact cognition and development in both typical and atypical populations. The vast literature on WM capacity development has revealed a nearly universal increase in capacity estimates over development and substantial variation in capacity estimates across task contexts within age groups. We contend that theoretical explanations focusing on single or separable mechanistic sources of children’s performance in WM tasks or development changes in capacity limits cannot provide an adequate account of the full pattern of performance shown in Tables 1– 4.

We began by presenting five questions that arose from our survey of the literature on estimates of capacity development, which raised possible explanations for the wide variation in results in Tables 1– 4. As we have described above, we reject these potential explanations and propose a new approach to studying WM capacity development. In summary, we do not believe that different tasks are tapping capacity in different ways, that difficult tasks are masking underlying competencies, or that capacity can only be understood by developing a single “best” paradigm to use for comparisons. Furthermore, we do not think that existing theories could account for the breadth of data by incorporating more causal mechanisms and/or being more specific. Rather, we argue that capacity does not exist in the way it has been traditionally conceptualized, but is an *emergent process within a dynamically coupled, self-organizing cognitive, and behavioral system*.

We propose that WM capacity should be construed as a dynamic *process* rather than a *property* of WM, and that we will gain better understanding of developmental change by first considering how the relevant cognitive and behavioral systems organize in service of tasks within and outside the laboratory, and second, by examining how changes in real-time dynamics create the developmental change we observe. We illustrated how Perone et al. (2011) used such an approach to provide insights into variation in capacity estimates across contexts and development improvement in performance during infancy. Furthermore, Simmering (2008) (2008, under review) showed how differences in the method of presentation and behavioral measures across paradigms could result in higher or lower capacity within the same individuals. Critically, performance was correlated across these tasks, supporting the claim that these tasks depend on the same underlying cognitive system.

Finally, we considered three significant challenges that arise when conceptualizing WM capacity as a dynamic process within an integrated system. To develop an effective systems theory of any cognitive process, not just WM capacity, we must specify the system components and how they operate across behavioral tasks while keeping a tight link between theories of how those processes work and the paradigms we use to test such processes. Within WM research more specifically, we urge theorists to integrate studies across verbal and visuo-spatial domains as well as simple and complex tasks, to arrive at a more complete understanding of the WM system’s self-organization. By confronting these challenges we believe that theories of WM capacity development will be able to expand beyond laboratory tasks and understand the role WM plays in the great variety of real world behaviors of interest across both typical and atypical populations.

## Conflict of Interest Statement

The authors declare that the research was conducted in the absence of any commercial or financial relationships that could be construed as a potential conflict of interest.

## Footnotes

^1^Note that we use the phrase “capacity estimate” to refer to data derived from behavioral studies, and “WM capacity” to refer to the psychological construct throughout this paper.

## Appendix

Here we provide a complete list of articles we reviewed for this paper, with a list of which experiments were included or excluded and why. First, the following papers have data included in Tables 1– 4. For all papers included, mean ages of children, along with either standard deviation or range, are reported when available. Second, we list papers that were entirely excluded, grouped by the reasons for exclusion.

Papers with data included:

1. Bayliss et al. (2005a). In a single experiment, 56 children (*M* age = 8.01 years, range = 7.01–9.00) participated in a four complex span tasks, two processing tasks, and two storage tasks. Results from the two storage tasks – digit span and Corsi block span – are reported in Tables 1 and 2. Results from the complex span tasks are reported in Table 4. For these tasks, processing and storage episodes were interleaved; both types of episodes could be verbal or visuo-spatial, resulting in four task types of the combined processing and storage types. Stimuli were nine colored squares that were either large or small. In the verbal processing task, children named a word commonly associated with the color (e.g., green = grass). In the visuo-spatial processing task, children searched for a target square (one of the large squares that included a beveled edge). The storage tasks were digit span (verbal) and Corsi block (visuo-spatial). List length (including both processing and storage episodes) increased after three successive correct trials; span scores were calculated as the average length on the last three correctly recalled trials.
2. Bayliss et al. (2005b). A single experiment included 40 children in each of three age groups at 6 years (*M* = 6.02, range = 5.08–6.07), 8 years (*M* = 8.04, range = 7.11–9.01), and 10 years (*M* = 10.03, range = 9.09–10.10). Children completed two sessions: in the first, they performed two tasks measuring processing efficiency and two measuring storage capacity; in the second session, two tasks measured complex span, two measured basic speed, and two measured rehearsal speed. Results from the two measures of storage capacity – digit span and the Corsi block task – are reported in Tables 1 and 2. The complex span tasks were as described above (2005), except only with the same domain (verbal versus visuo-spatial) across both processing and storage tasks; results are reported in Table 4.
3. Case et al. (1982). Experiment 1 tested 12 3-year-olds (*M* = 3.1, SD = 2.9 months), 10 4-year-olds (*M* = 3.10, SD = 2.5 months), 9 5-year-olds (*M* = 5.1, SD = 1.9 months), 9 6-year-olds (*M* = 6.1, SD = 3.2 months) on a word span task, which is reported in Table 1. Experiments 2 and 4 tested adults and are therefore excluded. Experiment 3 tested 84 children, 12 each at grades K (*M* = 6.0 years, SD = 1.6 months), 1 (*M* = 6.9 years, SD = 2.2 months), 2 (*M* = 7.8 years, SD = 2.5 months), 3 (*M* = 8.9 years, SD = 2.0 months), 4 (*M* = 9.9 years, SD = 0.85 months), 5 (*M* = 10.9 years, SD = 3.5 months), and 6 (*M* = 12 years, SD = 2.4 months), on a complex counting span task. In this task, children counted dots in sequential visual arrays, then held the results of these counts in memory; results are reported in Table 4.
4. Chi (1977). Experiment 1 tested six 5-year-olds’ memory for arrays of two to five faces, manipulating familiarity across conditions within subjects. Responses were scored twice, once counting faces that maintained the correct spatial layout, and once with layout irrelevant; both are reported in Table 2. For the conditions where spatial layout was irrelevant in the responses, scores increased with set size. Estimates reported here correspond to the highest set size tested (five faces).
5. Cowan et al. (2011). A single experiment included 90 participants, 30 in each of three age groups: third-graders (*M* age = 7.67 years, range = 6.3–9.0), sixth- to seventh-graders (*M* age = 12.42, range = 11.2–13.5), and adults (whose results are excluded from Table 4). Participants viewed a 4 × 3 grid on a computer screen in which four objects were sequentially presented in different squares of the grid (500 ms per item, with 500 ms delays between items). Two objects were circles and two were triangles; each object could take on one of nine colors without replacement across objects within a trial. Following the presentation of the to be remembered items, a probe display showed the grid with a single item (circle or triangle) that could match one of the memory items in both color and location, just color, or just location. The participant’s task was to indicate whether the item matched exactly or one or both color and location were different. Before the task, half of the participants were told to attend to only circles, the other half to only triangles; both types of items were tested for all participants, but most trials tested the shapes to be attended. Only results from attended items are reported in Table 4, as performance on unattended items was not reported as capacity. Across trial blocks, participants were instructed to remain silent during item presentation, to name the color of each item following its presentation, or say “wait” following each items’ presentation. These three trial types are reported separately in Table 4. Capacity was estimated using the equation from Cowan (2001).
6. Cowan et al. (2005). Experiment 1 tested 37 third-graders (*M* age = 8.79 years, SD = 5.36 months), 37 fifth-graders (*M* age = 10.72 years, SD = 5.85 months), and 63 adults in seven tasks across two sessions. Experiment 2 tested 29 second-graders (*M* age = 8.29 years, SD = 4.97 months,), 36 fourth-graders (*M* age = 10.10 years, SD = 7.54 months), 33 sixth-graders (*M* age = 11.92 years, SD = 5.81 months), and 29 adults in nine tasks across two session. Tables 1 and 2 include results from auditory sequences span (sine-wave tones; Experiment 2 only), digit span, and visual arrays (color change detection), with results averaged across experiments for 8- and 10-year-olds children (adults’ data are excluded). Table 4 includes four complex tasks, again with results averaged across experiments for 8- and 10-year-olds children (adults’ data are excluded): counting span (described above; Case et al., 1982), listening span, running memory span, and memory for ignored speech. In the listening span task, children listened to spoken sentences and judged each as true of false as well as remembering the final word of the sentence for later recall. Capacity is estimated as the number of final words the child later recalled. The running span task presents auditory digits at 0.25 s each, for lists of 12–20 items. At the end of a list, participants reported the last five, six, or seven items from the list, in forward order. This task differs from standard digit span because participants do not know when the list will end, thus they do not know which digits will need to be reported. In the ignored speech task, children listened to auditory digits while completing a silent game in which they had to select rhymes from among pictures presented on a computer screen. At random intervals, a trial of the rhyme game was replaced by instructions to recall as many auditory digits as possible.
7. Cowan et al. (2006a). Experiment 1 tested 18 children (*M* age = 8.51 years, SD* *= 3.62 months) and 18 adults in a digit span task with varying rates of stimulus presentation. These conditions did not significantly affect the number of items recalled, so children’s averaged results are presented in Table 1; adults’ results are excluded.
8. Cowan et al. (2006b). A single experiment tested 52 children (*M* age = 10.83 years, SD = 5.18 months) and 52 adults in six tasks: (1) an auditory digit span test, (2) a visual letter span test, (3) a dual-task to measure capacity, in which auditory digits and visual letters were presented together, (4) a visual array task to measure capacity, (5) a vocabulary test, and (6) a pattern-analysis test. Children’s results from tasks 1, 2, and 4 are reported in Tables 1 and 2; task 2 is repeated across tables, as it is unclear whether children recoded the stimuli verbally. Task 3 included conditions in which children were instructed to preferentially attend to or ignore one modality or the other; results are reported in Table 4.
9. Cowan et al. (2010). A single experiment included 90 participants, 30 in each of three age groups: first- to second-graders (range = 7–8 years), sixth- to seventh-graders (range = 12–13 years), and adults (whose results are excluded from Table 4). The task was similar to that described for Cowan et al. (2011) above with third exceptions. First, the memory items were presented simultaneously rather than sequentially. Second, rather than attending to only one shape across all trials, participants were instructed to attend to one or both across trial blocks. Third, the proportion of probe trials that tested the attended versus unattended items varied. In “1-shape” blocks, all items in the memory array were the same shape as each other (the attended shape); thus all probe items had to be the attended shape. In “100–0%” blocks, half of the items in the memory array were the attended shape and half were the unattended shape, and the probed item was always the attended shape. In the “80–20%” blocks, memory arrays were again half attended and half unattended, but probe items were the attended shape on 80% of trials (with 20% probing the unattended shape). Similarly, in the “50–50%” blocks, the attended and unattended shapes were equally likely to be probed. Capacity was estimated separately for each trial block, as well as for different numbers of memory items (two or three per shape) using the equation from Cowan (2001). Table 4 reports capacity estimates separately for trials with two versus three shapes and each trial type; note that 20% trials were the trials probing the unattended shape in the 80–20% block.
10. Cowan et al. (1999). A single experiment included 24 first-graders (*M* age = 7.41 years, SD = 3.73 months), 24 fourth-graders (*M* age = 10.24 years, SD = 5.22 months), and 24 adults (whose results are excluded from Tables 1 and 4). Participants were tested on four tasks: a digit span pretest, an auditory-only task, a visual-only task, and a visual-auditory task. For the pretest, digits were presented at one per second beginning with a list length of three digits. After the participant correctly recalled any list(s) at this length, the length was increased by one digit; trials proceeded in this manner until the participant could not correctly recall any lists at that length, or a maximum length of nine digits was reached. Span was considered the longest list length repeated correctly, and is reported in Table 1. Each participant’s span estimate from this task was used to design span-relative list lengths for subsequent trials: span, span-1, span-2, and span-3.
  The auditory-only task was similar to the pretest, except that participants were tested on these span-relative lengths. These results are reported as attended digits in Table 1. From these results, the authors also compared performance across participants on a single list length to, see if the length of the presented list constrained performance; these results were reported as mean number correct, rather than a span estimate, and are therefore excluded from Table 1. For the visual-only task was a picture naming task that did not yield a span measure; it was included to familiarize participants with the second part of the task required for the visual-auditory task. In this dual-task, participants heard the same lists as in the auditory-only task but were instructed to ignore it and attend instead to the visual picture naming task. Results from this task are reported in Table 4. Results for attended and unattended lists were note reported in the text, but had to be estimated from Figure 1; we estimated span as the highest number correct across span lengths.
11. Cowan et al. (2006c). Experiment 1 tested 32 third-graders (*M* age = 8.63 years, SD = 0.36), 32 sixth-graders (*M* age = 11.93 years, SD = 0.36), and 32 college students in five tasks measuring (1) verbal-to-spatial mapping, (2) spatial span, (3) name span, (4) counting span, and (5) running span. Results were reported as both the maximum span length children got correct and the sum of span lengths; given that children completed different numbers of trials, we report only the maximum. Spatial and name spans are reported in Tables 1 and 2, with adults excluded. Note that, unlike most verbal span tasks, in the name span task stimuli were presented visually and children were tested by presenting all possible names and asking them to arrange the ones that had been presented in the correct order. Results of the verbal-to-spatial mapping task were not reported as capacity estimates and are therefore not included in Table 4. The counting and running span tasks were similar to those described above (respectively: Case et al., 1982; Cowan et al., 2005), and are reported in Table 4. Experiment 2, which tested only college students, is excluded.
12. de Ribaupierre and Bailleux (1994). A longitudinal study included 120 children (30 per age group) aged 5, 6, 7, 8, and 10 years at the study onset; children were tested once each year, within 2 months of their birthday, over 5 years. Results are based on the 100 children who contributed data each year (*n* = 27, 22, 28, and 23, respectively). At each test session, children completed two tasks assessing visuo-spatial with a “Mr. Peanut” figure that could have colored dots appearing at specified locations over his body. In the first task, dots were the same color and children had to remember their locations. In the second task, each dot was a different color, and children had to remember both locations and color-location bindings. Results of these tasks are reported in Tables 2 and 4, respectively; note that results are averaged across cohorts when they were tested at the same age (i.e., the mean for 8 years includes data from children who began the study at 5, 6, 7, and 8 years).
13. de Ribaupierre et al. (1989). A single experiment included 192 children: 42 each at 5, 6, 8, and 10 years, and 12 each at 7 and 9 years; all children were tested within 2 months of their birthdays. The procedure was the same as in de Ribaupierre and Bailleux (1994), testing memory for locations or color-location bindings in the “Mr. Peanut” task; again, results are reported in Tables 2 and 4, respectively.
14. DeMarie and Ferron (2003). A single experiment tested 185 children across multiple sessions; ages were calculated on the first day of testing. The final sample included 28 kindergarteners (*M* age = 5.58 years), 35 first-graders (*M* age = 6.92 years), 43 second-graders (*M* age = 8.08 years), 30 third-graders (*M* age = 9.0 years) and 43 fourth-graders (*M* age = 10.0 years). All children completed a battery of tasks measuring a variety of cognitive processes; of relevance here are only two word span tasks (animals or clothing, reported together) and one digit span task, which are included in Table 1.
15. Dempster (1981). This paper reviewed pervious research on capacity development, and presented mean data in separate figures for digit span, word span, and letter span. Table 1 includes estimates derived from these figures. Note that studies included in Dempster’s review are not otherwise included here.
16. Engle and Marshall (1983). A single experiment tested 24 first-graders (*M* age = 6.8 years, SD = 0.38), 24 sixth-graders (*M* age = 11.9 years, SD = 0.28), and 24 adults in a digit span task manipulating presentation time and grouping. Table 1 includes only children’s data from the “ungrouped” condition, separated by presentation rates.
17. Gilchrist et al. (2009). A single experiment tested 25 first-graders (*M* = 7.73 years, SD = 0.21), 26 sixth-graders (*M* = 12.43 years, SD = 0.39) and 24 adults. Participants were presented with lists of words organized in sentences (short or long) or random pseudo-sentences. Because the primary goal of presenting words organized as sentences was to encourage chunking words into sentences, which then become the unit of memory, these conditions are excluded from Table 1; adults’ results are also excluded.
18. Halford et al. (1994). Experiment 1 tested 24 9-year-olds (*M *= 9.33, range = 8.83–9.83). Children were shown six cards containing familiar words at the beginning of the trial and asked to read them aloud from left to right. Next, they performed an unrelated task that required either subtracting or reading numbers (between subjects). Finally, they were presented with the six cards from the beginning of the trial and asked to arrange them in the original order. The mean number correct across trials was reported as span, and is included in Table 4. Experiment 2 tested 40 children (*M* = 9.83 years, range = 8.42–11.67) in a similar task: children were presented with an auditory list of digits to begin the trial, then given a transitive inference problem as the secondary task (with a between subjects manipulation of the problem presentation). After solving the problem, they were asked to repeat back the digits in the original order. The number of digits was increased following correct trials to reach a span estimate, which is reported in Table 4. Experiment 3 tested 16 5-year-olds (*M* = 5.5, range = 5.0–5.92), 16 9-year-olds (*M* = 9.0, range = 8.58–9.5), and 16 12-year-olds (*M* = 12.42, range = 12.0–12.92) in digit span tasks with and without a counting dual-task; results without the dual-task are reported in Table 1, and results with the dual-task are reported in Table 4. Experiment 4 tested 20 8-year-olds (*M *= 8.33, range = 8.0–8.92) and 20 12-year-olds (*M *= 12.33, range = 12.0–13.17) in three types of dual-tasks, within subjects: counting forward, counting backward, and listening to a story; results are reported in Table 4.
19. Hamilton et al. (2003). Study 1 tested 30 5- to 7-year-olds children (*M* = 6.5 years, SD = 0.95), 30 8- to 10-year-olds children (*M* = 9.4 years, SD = 0.92), 30 11- to 13-year-olds children (*M* = 12.1 years, SD = 0.7), and 30 adults in visual span, spatial span, speech articulation, and verbal fluency tasks. Only children’s results from the visual and spatial span tasks are reported in Table 2. Study 2 tested 78 6- to 9-year-olds children (*M* = 7.9 years, SD = 1.07) and 39 adults in the visual and spatial span tasks with five conditions manipulating interference during the delay period. Children’s results from only the no-interference/control condition are reported in Table 2.
20. Hitch et al. (1988). Experiment 1 included 36 5-year-olds (*M* = 5.5, range = 5.0–6.0) and 18 10-year-olds (*M* = 10.5, range = 10.17–11.08). Children viewed sequences of line drawings (three for 5-year-olds, five for 10-year-olds), then reported the item name as the experimenter pointed to the locations where the drawings had been presented. All children completed three types of tasks: similar (in which pictures visually resembled one another), long names (three syllables, not visually similar), and control (monosyllabic, not visually similar). Half of the 5-year-olds were told to name the pictures when they were first presented, while the remaining 5- and 10-year-olds were instructed to remain silent. Results are included in Table 1.
  Experiment 2 included 24 5-year-olds (*M* = 5.2, range = 4.83–5.92) and 24 10-year-olds (*M* = 10.75, range = 10.25–11.25). This procedure was identical to the control condition from Experiment 1 except that children were asked to name the drawing immediately after its presentation, as soon as it was out of view. Additionally, on half of the trials children were asked to recall in the same order as the items were presented, while the other half of trials were recalled in the opposite order; Table 2 includes the forward repetition, Table 3 includes the backward repetition. In this experiment, results were presented in a figure as percent correct across serial order, from which we estimated the average number correct for each age group. Experiments 3, 4, and 5 tested the effects of interference and are not included here.
21. Hitch et al. (1989). Experiment 1 included 18 5-year-olds (*M* = 5.25 years, range = 4.75–5.67) and 36 11-year-olds (*M* = 10.83 years, range = 10.33–11.25); half of the 11-year-olds were assigned to an articulatory suppression condition, which is excluded from Table 2. Children remembered sets of drawings that were visually similar, had phonemically similar names, or were dissimilar in both ways (control). Experiments 2 and 3 tested effects of retroactive interference and did not report mean number correct/capacity estimates, and are therefore excluded from Table 2.
22. Hulme et al. (1984). Experiment 1 included nine participants in each of four age groups: 3–4 years (*M* = 4.08 years, range = 3.58–4.25), 7–8 years (*M* = 7.80 years, range = 7.17–8.08), 10–11 years (*M* = 10.88 years, range = 10.33–11.5), and adults. Experiment 2 included 18 8-year-olds (*M* = 8.47 years, range = 8.17–8.83) and 18 10-year-olds (*M* = 10.63 years, range = 10.08–10.92). In both experiments, participants’ speech rates were recorded and compared with their mean number of words recalled per list (six-word lists for adults, five-word lists for older children, four-word lists for younger children). Words were short (one syllable), medium (two syllables), or long (three or four syllables), which yielded significantly different results, with higher spans for shorter words. Note that the 10- to 11-year-olds from Experiment 1 are listed as 11 years in Table 2 to differentiate from the 10-year-olds in Experiment 2.
23. Huttenlocher and Burke (1976). One experiment included 90 preschool children (*M* age = 4.6 years), 60 first-graders (*M* age = 7.0 years), 60 third-graders (*M* age = 9.2 years), and 60 fifth-graders (*M* age = 11.0 years). Children were asked to repeat lists of digits, preserving presentation order. Temporal grouping was manipulated across lists (within subjects), and participants were randomly assigned to one of three types of sound pattern, prosodic, melodic, or monotone; results reported in Table 1 are averaged across sound pattern conditions.
24. Hutton and Towse (2001). A single experiment tested 29 8-year-olds (*M* = 7.58 years, range = 7.08–8.08) and 25 10-year-olds (*M* = 10.75 years, range = 10.25–11.25) children in a digit span task (forward and backward recall, with and without articulatory suppression) and a counting span task (forward and backward recall), followed by a series of “ability” measures (i.e., tests of intelligence and aptitude). Results from forward recall without suppression are reported in Table 1; results from backward recall without suppression are reported in Table 3. All other results are reported in Table 4.
25. Imbo and Vandierendonck (2007). A single experiment included 21 fourth-graders (*M* age = 10.0 years), 21 fifth-graders (*M* age = 11.08 years), and 21 sixth-graders (*M* age = 12.17 years). Children completed a series of tasks assessing math skills, executive functioning, processing speed, and memory span; only the span task (for digits) is reported in Table 1.
26. Isaacs and Vargha-Khadem (1989). A single experiment included 288 children, divided into groups of 32 at each of nine age levels between 7 and 15 years (inclusive; no further details of age were given). Children completed digit span tasks, forward and backward, and the Corsi block task, forward and backward. In both tasks, the longest string of digits or blocks which the child recalled correctly at least once was recorded as that child’s span. Forward versions of the tasks are reported in Tables 1 and 2; backward versions of both are reported in Table 3.
27. Kyttälä (2008). A single experiment included 45 ninth-graders (age range = 15–16 years) who were selected to form three groups of 15 each: low math/normal reading ability; low math/low reading ability; and normal ability controls. All students completed three “passive” memory tasks – the visual patterns task, Corsi block task, and “little houses” task (requiring recall of house forms) – and two “active” tasks that required either drawing from memory or making judgments of linearity from memory. The three passive tasks are reported in Table 2, separated by student group.
28. Logie and Pearson (1997). A single experiment tested 62 5-year-olds (*M* = 5.75 years, SD = 3.3 months), 44 8-year-olds (*M* = 8.83 years, SD = 3.7 months), and 40 11-year-olds (*M* = 11.83 years, SD = 3.9 months) in visual and spatial tasks. For the visual task, children were presented with a 10- or 12-square matrix in which half of the squares were filled; they were then tested on both recognition (yes/no response to a second matrix) and recall (fill in blank matrix) of the pattern. The spatial task was a variation of the Corsi block task, in which nine identical blue blocks were placed in front of the child, and the experimenter tapped out a sequence of blocks. Children were again tested on both recognition (yes/no response to a second tapping sequence by the experimenter) and recall (tapping blocks themselves) of the sequence.
29. Luciana et al. (2005). A single experiment tested 106 9- to 17-year-olds (*M* = 13.52 years, SD* *= 2.82) and 27 18- to 20-year-olds (excluded from Table 2) in a battery of working memory tasks. Only two tasks yielded span/capacity estimates, spatial span forward and backward, based on the Corsi block task; forward span results are reported in Table 2, backward span in Table 3.
30. Miller and Vernon (1996). A single experiment tested 109 children between 4 and 6 years of age (*M* = 5.51 years, SD = 0.85). Children’s intellectual ability was measured and compared to reaction times in a speed of processing task and a series of working memory tasks. The working memory tasks are reported in Tables 1 and 2 and included two color memory tasks (sequential and simultaneous presentation), two shape memory tasks (sequential and simultaneous presentation), and a tone memory task. Capacity was calculated as the highest sequence/array sizes at which the child performed correctly on both trials.
31. Morra (1994). Experiment 1 tested 191 children ranging in age from 6.0 to 10.92 years; results were reported in groups for each year of age, although mean ages for each group were not reported. This study included three sessions with 16 tasks total, 8 of which yielded span estimates: forward digit span, backward digit span, forward Corsi block, backward Corsi block, forward word span, backward word span, counting span (cf. Case et al., 1982), and the Mr. Cucumber task (comparable to the Mr. Peanut task with color-location bindings as described above; de Ribaupierre and Bailleux, 1994). Results from the simple/forward tasks were not reported as span estimates, but were only used for correlation and factor loading analyses. Results from backward tasks are included in Table 3, and results from the counting span and Mr. Cucumber tasks are reported in Table 4. Experiment 2 tested 124 children ranging in age from 5.83 to 9.75 years; results were reported for 6- (*M* = 6.25), 7- (*M* = 7.42), 8- (*M* = 8.42), and 9- (*M *= 9.33) year-olds. Participants completed a subset of the tasks from Experiment 1, including forward and backward digit span, counting span, and Mr. Cucumber. Reported results (all but forward digit span) are included in Tables 3 and 4, averaged with results from Experiment 1 for all age groups but 10-year-olds (as this age group was not included in Experiment 2).
32. Morra and Camba (2009). A single experiment tested 58 third-graders (age range = 8.08–9.33 years), 51 fourth-graders (age range = 9.17–10.33 years), and 52 fifth-graders (age range = 10.17–11.25 years) in 13 tasks across two sessions. Four of these tasks yielded capacity estimates: Mr. Cucumber (similar to the “Mr. Peanut” task described above; see de Ribaupierre and Bailleux, 1994), counting span, forward digit span, and backward digit span. These four tasks are included in Tables 2 (as pattern recall), 4, 1, and 3, respectively. For all tasks, means were not reported separately by age group.
33. Mutter et al. (2006). A single experiment tested 72 preschool children (*M* age = 3.92 years, range = 2.67–5.33) in a variation of the “Mr. Peanut” task described above (see de Ribaupierre and Bailleux, 1994). The Figure had between one and six colored body parts, with three trials to each level; children indicated which parts had been colored after a 2 s delay. Capacity was calculated by awarding one-third point for each correct trial. The task ended when participants answered incorrectly for all trials at one level.
34. Nichelli et al. (2001). A single experiment included 275 children, divided into seven age groups: 31 5- to 6-year-olds (range = 64–83 months), 50 7-year-olds (84–95 months), 33 8-year-olds (96–107 months), 47 9-year-olds (108–119 months), 53 10-year-olds (120–131 months), 36 11-year-olds (132–143 months), and 23 12-year-olds (144–162 months); mean ages and standard deviations were not reported. All children completed a spatial span (Corsi) task, and most also completed a verbal (word) span task (*n* = 31, 40, 29, 38, 46, 31, and 23, respectively by age). The verbal task included an immediate span estimate as well as a learning component; only immediate span is reported in Table 1.
35. Noël (2009). A single experiment included 80 children, 38 in their second year of Belgian kindergarten (*M* age = 4.42 years, SD = 3.9 months, range = 3.92–5.0 years), and 42 in their third year of Belgian kindergarten (*M* age = 5.42 years, SD = 3.9 months, range = 4.83–6.08 years). Children’s memory capacity was assessed in addition to counting and addition skills, non-verbal intelligence, and general vocabulary. The capacity tasks included two verbal span tasks, one for monosyllabic words, one for food and animal names, and the Corsi block task. Recall performance did not differ across the two types of words, so results are averaged together in Table 1.
36. Orsini et al. (1981). A single experiment included 1113 children between the ages of 4 and 10 (no further detail given); children were classified as “town” or “country” dwelling as an index of cultural background. All children completed the Corsi block task and a word span task. Results were presented separately by gender and by cultural background, but are averaged together for Tables 1 and 2.
37. Ottem et al. (2007). Study 1 tested 65 third-graders (*M* age = 8.5 years, SD* *= 0.42) and 35 seventh-graders (*M* age = 12.5 years, SD = 0.88) on tests of language performance (serial recall of words) and a non-verbal test of cognitive performance. Lists of words were classified as being distinct or similar within lists. Span was computed as the longest list that the child recalled in the correct order; testing was terminated when the participant made errors on three consecutive lists. Study 2 tested 934 children (range = 6–16 years) in two language skill tasks and a shortened version of the serial recall task, again classifying lists as distinct or similar. Results were reported separately for age groups divided by year (i.e., 6–7 years, 7–8 years, etc.) without specifying the mean ages per group. Study 3 included 29 3-year-olds (*M* = 3.59 years, SD = 0.34), 50 4-year-olds (*M* = 4.53 years, SD = 0.27), and 44 5-year-olds (*M* = 5.47 years, SD = 0.28). Children completed a shorter version of the serial recall task, again with distinct or similar words within lists, as well as two tests of receptive language performance. Results from the list recall tasks are presented in Table 1, averaged across Studies 1 and 2 for 8- and 12-year-olds.
38. Palmer (2000). Experiment 1 included 12 3-year-olds (*M* = 3.58 years, range = 3.08–4.08), 26 6-year-olds (*M* = 6.00 years, range = 5.42–6.33), 28 7-year-olds (*M* = 7.00 years, range = 6.42–7.33). Children were randomly assigned to one of two conditions, control or verbal recoding. All children were shown 12 trials consisting of series of pictures (lists of three for 3-year-olds, lists of four for 6- and 7-year-olds), and those in the verbal recoding condition were asked to name them aloud during encoding. Of the 12 trials, 4 included photos of visually similar objects, 4 included photos of phonologically similar objects, and 4 included dissimilar (control) objects. Results are reported as the mean number of objects identified in the correct serial position. Experiment 2 included 38 5-year-olds (*M* = 5.29 years) and 42 6-year-olds (*M* = 6.25 years) who were tested three times in each of three consecutive years (at final test, *n* = 34, *M* = 7.29 years, and *n* = 39, *M* = 8.25 years, respectively). Children were not instructed on whether to verbally encode the objects, and were tested on the same 12 trials from Experiment 1 (all list lengths of four). Results are reported as the mean number of objects identified in the correct serial position.
39. Pickering et al. (1998). A single experiment tested 28 5-year-olds (*M* = 5.7 years, SD = 2.59 months) and 31 8-year-olds (*M* = 8.8 years, SD = 2.15 months) on serial recall of letters, digits, and blocks. For digit span, only number of correct lists was reported (not span/capacity estimates), so these results are excluded from Table 1. For the letter and block tasks, estimates from two different list lengths are reported for each group; the averages across lengths are included in Tables 1 and 2.
40. Riggs et al. (2006). One experiment included 20 children in each of three age groups: 5-year-olds (*M* = 5.54 years), 7-year-olds (*M* = 7.25 years), and 10-year-olds (*M* = 10.63 years). All children completed a color change detection task in which a memory array included one to five colored squares, followed by a short delay and presentation of a test array with the same number of items. Colors in the test array were either all identical to the memory array (“same” trials) or one color had changed (“different” trials). Capacity was estimated for each set size using Pashler’s (1988) formula, and was averaged across set size for the values reported in Table 2.
41. Russell et al. (1996). Experiment 1 included three groups of children: 33 with autism diagnoses, 33 with moderate learning difficulties, and 33 typically developing (*M* age = 6.28 years, SD = 1.19); only results from typically developing children are reported in Table 1. All children completed a verbal span task with both short (one syllable) and long (three syllable) words, using two response types: verbal, in which they reported the list back in sequence; and non-verbal, in which they pointed, in sequence, to pictures corresponding to the words in the list. Span was estimated as the longest list length at which a child had been correct on at least two of the three trials, with a further half point credited if the child was correct on one of the three trials at the next list length. Results from these span tasks were compared to speed of articulation for both short and long words.
  Experiment 2 tested similar groups, with 22 typically developing children (*M* age = 6.85 years, SD = 0.5) in three complex span tasks, within subjects: counting span, sum span, and the odd-man-out task; each had a “simple” and “complex” form. For the counting span task, the simple form presented dots in familiar arrangements (i.e., as on dice), but in unsystematic arrangements for the complex form. In the sum span task, children were presented visually with simple addition problems; in the simple form, the answers were provided with the problem, and in the complex form children had to generate the answer on their own. The odd-man-out task included a series of cards with three dots, one of which was a different than the other two. Children were required to point to the unique dot and remember its position (within a 3 × 4 grid) across cards to report, in order, at the end. In the simple form, the unique dot was black on every trial; in the complex form, the dots had different patterns on every card. Results from these tasks are reported in Table 4.
42. Simmering (2012). A single experiment tested 14 3-year-olds (*M* = 3.39 years, SD = 2.35 months), 14 4-year-olds (*M* = 4.21 years, SD = 1.77 months), 28 5-year-olds (*M* = 5.15 years, SD = 3.11 months), and 28 7-year-olds (*M *= 7.48 years, SD = 3.08 months) in a color change detection task. All of the 3- and 4-year-olds and half of the 5- and 7-year-olds completed a modified version of the task in which one to five colored squares were presented within a gray rectangular frame, labeled as “cards” to facilitate younger children’s understanding of the task. The remaining 5- and 7-year-olds completed a replication of the task from Riggs et al. (2006). In both tasks, capacity was estimated for each set size using Pashler’s (1988) formula; each child’s highest estimate across set sizes was then averaged across children within each age group to arrive at estimates reported in Table 2. There were no significant differences between the modified and replication versions, so these estimates were averaged together in Table 2.
43. Towse and Hitch (1995). Experiment 1 tested only adults and is therefore excluded. Experiment 2 tested 76 children, divided into four age ranges: 6-year-olds (*M* = 5.92, range = 5.42–6.42), 7-year-olds (*M* = 6.92, range = 6.5–7.42), 8-year-olds (*M* = 7.92, range = 7.5–8.42) and 10-year-olds (*M* = 10.75, range = 10.17–11.33). Children completed a counting span task (Case et al., 1982) with three trial types. On feature trials, cards included blue target (i.e., to be counted) squares and orange non-target (i.e., to be ignored) triangles. On conjunction trials, cards include blue target squares and blue non-target triangles. On feature-slow trials, cards were similar to the feature trials, but with larger numbers of items (i.e., 6–10 rather than 3–7). Span was measured as the number of counting results children retained across trials and reported correctly at the end of trials; note that counting errors were ignored, such that recalling an incorrect count was considered correct. Results are reported separately for trial types in Table 4.
44. Towse et al. (1998). Experiment 1 included 67 children divided into four age groups: 6-year-olds (*M* = 6.9, range = 6.3–7.2), 7-year-olds (*M *= 7.8, range = 7.3–8.1), 9-year-olds (*M *= 9.9, range = 9.4–10.2), and 10-year-olds (*M *= 10.5, range = 10.2–11.1). Children performed a counting span task similar to the feature condition described in Towse and Hitch (1995), with small and large number of items per card alternating within trials. Across trials, whether the trial began with a small versus large number was counter-balanced, and the final card in the sequence was always opposite (i.e., small first card, large final card). Results were reported separately for these trials types in Table 4. Experiment 2 included 55 children divided into three age groups: 8-year-olds (*M *= 7.11, range = 7.5–8.4), 10-year-olds (*M *= 10.1, range = 9.5–10.5), and 11-year-olds (*M *= 10.1, range = 10.5–11.5). Children were tested in an operation span task in which they performed a simple addition or subtraction problem and retained the result for later recall. The length of problems was varied (short = two terms, intermediate = three terms, long = four terms), and results were reported separately depending on the length of the final problem in the set, as shown in Table 4. Experiment 3 tested 65 children divided into three age groups: 8-year-olds (*M *= 8.4, range = 7.1–8.9), 9-year-olds (*M *= 9.3, range = 8.1–9.8), and 10-year-olds (*M *= 10.3, range = 9.8–10.8). Across two counter-balanced sessions, children performed an operation span task as in Experiment 2, as well as a reading span task in which children had to read a sentence and fill in a blank for the final word; the final words were then retained for recall. As in the other tasks, the length of sentences was manipulated. Results are reported in Table 4 (with operation span results averaged across Experiments 2 and 3 for 8- and 10-year-olds).
45. Towse et al. (2002). Experiments 1 tested 7- to 17-year-olds children in an interpolated digit span task (requiring arithmetic between items to be remembered) but did not report span estimates. Similarly, Experiment 2 tested 8- and 10-year-olds children in an interpolated word or digit span task (requiring lexical decision tasks between items to be remembered) but did not report span estimates. Experiment 3 included 25 children (*M* age = 10.0 years, range = 9.67–10.5) were given four digits to recall on each trial, with one of two interpolated processing tasks (between participants). In the two-choice task, children reported either the parity of a number (even/odd) or the accuracy of an arithmetic sum for the interpolated task. In the four-choice task, participants were required to consider both parity and accuracy in conjunction. Results were reported as the number of digits (out of four) correctly reported in order at the end of each trial with short (6 s) versus long (15 s) delays resulting from the interpolated task, and are included in Table 4.
46. Vicari et al. (2003). Experiment 1 included 193 children divided into the following age groups: 23 5-year-olds (*M* = 5.5 years, SD = 0.3), 34 6-year-olds (*M* = 6.5 years, SD = 0.3), 38 7-year-olds (*M* = 7.5 years, SD = 0.4), 39 8-year-olds (*M* = 8.42 years, SD = 0.3), 32 9-year-olds (*M* = 9.58 years, SD = 0.4), and 27 10-year-olds (*M* age = 10.42 years, SD = 0.3). All children completed both spatial and visual span tasks. For the spatial task, a grid of nine blocks (similar to the Corsi task) appeared on a computer screen, and an abstract geometric shape appeared on one block, disappeared, then reappeared on another block, and so on to comprise spans of one to six blocks. Children reproduced the sequence of blocks on which the shape had appeared. For the visual task, a series of one to six non-definable geometric figure appeared in the middle of the computer screen, separated by a short delay. For test, a set of nine figures appeared and the participant indicated which matched the original figure(s) in the order of presentation. Experiment 2 used the same task with children with William Syndrome; these results are excluded.
47. Wagner and Jackson (2006). A single experiment included 120 children, 40 each from kindergarten (*M* age = 6 years SD = 0.39), first grade (*M* age = 7 years, SD = 0.30), and third grade (*M* age = 9 years, SD = 0.36). Children were randomly assigned to two “scanning” groups, in which they were instructed to use different visual scanning strategies to encode items for a span task using picture communication symbols. Pictures were presented in a 4 × 3 grid, and participants were required to recall which picture was where. Span was estimated as the highest number of pictures correctly retrieved across trials including 2–10 pictures, irrespective of location errors. Results, averaged across scanning conditions, are reported in Table 2 (note that the authors reported results as “word span,” but we chose to include them with other visuo-spatial tasks because the stimuli and responses used pictures rather than verbal words).
48. Wilson et al. (1987). Four groups of 18 children each were tested in a single experiment: 5-year-olds (*M* = 5.33 years, SD = 2.47 months), 7-year-olds (*M* = 7.5 years, SD = 3.46 months), 11-year-olds (*M* = 11.42 years, SD* *= 3.4 months), and adults (data excluded). Participants completed a pattern span task over retention intervals of 2 or 10 s, tested with recognition. An additional condition with interference during the 10 s retention interval is not included in Table 2.
49. Wynn and Coolidge (2009). A single experiment tested 34 high school students (*M* age = 16.0 years, range = 15–17) in two tests of phonological capacity – digit span forward and backward – and a test of theory of mind. The span tasks continued until participants failed to recall one string correctly; span was estimated as the largest number of digits recalled, separately for forward and backward recall. Results from the forward and backward span tasks are included in Tables 1 and 3, respectively.
50. Zuber et al. (2009). A single experiment included 130 first-graders (*M* age = 7.33 years, SD = 7.1 months, range = 6.4–8.8 years). Children completed three tasks measuring verbal memory (letter span), visuo-spatial memory (Corsi block), and central executive (backward versions of other tasks), for comparison with performance on number word transcoding. The forward versions of the tasks are reported in Tables 1 and 2; in both cases, the maximum list length at which a child correctly recalled two lists was recorded as that child’s span. Backward versions are reported in Table 3.

The remaining papers were reviewed but excluded entirely from Tables 1 and 2, for reasons described below.

1. A number of papers included span- or capacity-related tasks, but reported only number/percent correct, rather than capacity estimates: Alloway et al. (2005), Alloway et al. (2009), Alloway et al. (2006), Alloway et al. (2004), Andersson (2010), Ang and Lee (2010), Arsalidou et al. (2010), Cowan et al. (2010), Das (1985), Engle et al. (1991), Engel et al. (2008), Gathercole and Pickering (1999, 2000), Gathercole et al. (2004), Grimley and Banner (2008), Holmes et al. (2008), Kaufman (2007), Kyttälä et al. (2003), Marcovitch et al. (2010), Nevo and Breznitz (2011), Nutley et al. (2010), Oakhill and Kyle (2000), Polderman et al. (2006), Reuhkala (2001), Walker et al. (1994).
2. Some studies computed capacity estimates for use in correlations, but did not report means: Fry and Hale (1996), Klingberg et al. (2002), Lehto (1996), Swanson and Berninger (1996), Whitebread (1999).
3. Other studies tested the same group of children repeatedly in order to assess learning: Swanson (1996, 1999, 2003).
4. Tasks designed for use with infants and toddlers rely on behavioral measures such as looking preferences or duration of searching behavior, which may involve processes in addition to those involved in tasks designed for older children and adults: Barner et al. (2007), Feigenson and Carey (2003, 2005), Káldy and Leslie (2005), Oakes et al. (2011), Oakes et al. (2009), Oakes et al. (2006), Rose et al. (2001), Ross-Sheehy et al. (2003, 2010), Zosh et al. (2011).
